# Patient’s Natural Killer Cells in the Era of Targeted Therapies: Role for Tumor Killers

**DOI:** 10.3389/fimmu.2017.00683

**Published:** 2017-06-12

**Authors:** Meriem Messaoudene, Alexandra Frazao, Pierre Jean Gavlovsky, Antoine Toubert, Nicolas Dulphy, Anne Caignard

**Affiliations:** ^1^INSERM U1015, Gustave Roussy Cancer Campus, Villejuif, France; ^2^Gustave Roussy Cancer Campus, Villejuif, France; ^3^INSERM U1160, Institut Universitaire d’Hématologie, Hôpital Saint Louis, Paris, France

**Keywords:** tumour immunosurveillance, natural killer ligands, immune checkpoint inhibitors, BRAF inhibitor, AMLMDS, melanoma

## Abstract

Natural killer (NK) cells are potent antitumor effectors, involved in hematological malignancies and solid tumor immunosurveillance. They infiltrate various solid tumors, and their numbers are correlated with good outcome. The function of NK cells extends their lytic capacities toward tumor cells expressing stress-induced ligands, through secretion of immunoregulatory cytokines, and interactions with other immune cells. Altered NK cell function due to tumor immune escape is frequent in advanced tumors; however, strategies to release the function of NK infiltrating tumors are emerging. Recent therapies targeting specific oncogenic mutations improved the treatment of cancer patients, but patients often relapse. The actual development consists in combined therapeutic strategies including agents targeting the proliferation of tumor cells and others restorating functional antitumor immune effectors for efficient and durable efficacy of anticancer treatment. In that context, we discuss the recent results of the literature to propose hypotheses concerning the potential use of NK cells, potent antitumor cytotoxic effectors, to design novel antitumor strategies.

## Introduction

Natural killer cells have been known and actively studied for more than four decades. They were first described as large granular lymphocytes cytotoxic for various tumor cells without prior stimulation (1, 2). In addition to their cytolytic activity against neoplastic and virus-infected cells, NK cells also display immunomodulatory functions by their ability to release cytokines, like interferon-γ (IFNγ) and tumor necrosis factor-α (TNFα), and chemokines. NK cells represent 5–15% of blood lymphocytes. They are present in the bone marrow, liver, uterus, spleen, lungs, in mucosa-associated lymphoid tissues, thymus, and secondary lymphoid tissues (SLT) and are recruited in inflamed sites. In SLT, NK cells provide an early source of IFNγ and interact with dendritic cells to promote T helper cell type 1 responses (3).

Natural killer cells are now grouped in the system of innate lymphoid cells (4). These populations, mostly tissue resident and characterized by their capacity to produce high amounts of cytokines, constitute innate homologs of T helper cell (CD4) and cytotoxic T cell (CD8) subsets. ILCs are implicated in tissue homeostasis and autoimmune diseases. Their distribution and capacity to produce cytokines suggest that they may also be involved in the development or evolution of cancer. NK cells are considered as cytotoxic counterparts of ILC1, both depending on the T-bet transcription factor for their development.

Human NK cells, defined as CD45^+^/CD3^−^/CD56^+^ cells (5), are classically subdivided in two subsets based on the relative membrane expression of CD56 and CD16, the low-affinity receptor for the Fc portion of IgG (FcγRIIIA): CD56^dim^ NK cells that express high levels of CD16 mediate antibody dependent cell cytotoxicity (ADCC), whereas CD56^bright^ NK cells express no or low levels of CD16. These two subsets are present in different proportions in the different tissues. CD56^dim^ NK cells represent 90% of blood and splenic NK cells, while CD56^bright^ NK cells predominate over CD56^dim^ in the SLT [lymph nodes (LN) and tonsils] representing up to 90% of NK cells and also constitute the major NK subset in tissues. It is accepted that CD56^bright^ NK cells are less mature than CD56^dim^ NK cells and display an immunoregulatory function, secreting high amounts of IFNγ and TNFα. CD56^dim^ NK cells represent mature NK cells with a high cytotoxic activity (6).

The activation of NK cells is tightly regulated by a balance between activating and inhibitory signals delivered through engagement of numerous activating and inhibitory receptors with ligands on the target cell. Natural cytotoxicity receptors (NCRs), such as NKp46 and NKp30, are expressed by resting NK cells while NKp44 is induced after activation by cytokines, such as IL-2 and IL-15 (7, 8). The NCRs are implicated in the lysis of various tumor cells (9). The activating NK group 2 member D (NKG2D) receptor is expressed by most circulating NK cells and binds the stress-induced MHC-class I polypeptide-related sequence (MIC)-A/B molecules and UL16-binding proteins 1–6 (ULBP1–6) (10). DNAX accessory molecule-1 (DNAM-1) binds Nectin family molecules CD155 and CD112.

Natural killer cell activation is efficiently controlled by specific inhibitory NK receptors binding human leukocyte antigen of class I (HLA-class I) molecules. The C-type lectin CD94/NKG2A receptor binds HLA-E molecules (11) sensing the global HLA-class I molecules on the target while killer Ig-like receptors (KIRs) bind classical HLA-class I molecules, including HLA-C, HLA-Bw4, and some HLA-A alleles.

## NK Cells in Tumor Immunosurveillance

A link between NK cell function and cancer development was reported in a Japanese 11-year follow-up study including 3,625 patients in which cancer incidence was negatively correlated with blood NK-mediated cytotoxicity (12). Authors further showed that individuals with particular NKG2D haplotypes, HNK1/HNK1 haplotype (correlated with high NK activity) had a decreased risk of cancer compared to those with an LNK1/LNK1 haplotype (correlated with low NK activity) (13).

Additional results including ours showed the impact of NCR transcripts in the evolution of melanoma, lung cancers, and gastrointestinal stromal tumors (GIST) patients (14–16). High NKp46 correlated with better survival in metastatic melanoma patients and particular profiles of NKp30 isoforms was associated with better outcome and response to treatment in GIST patients.

The cancer immunoediting process (17) resumes cancer progression in three phases. In the elimination phase, immune cells and among them NK cells eradicate developing tumor cells. During the equilibrium phase, the immune system may select tumor variants with less immunogenicity gradually leading to the tumor escape phase and tumor progression. It is considered that most tumors at diagnosis are in the phase of immune escape associated with functionally altered tumor infiltrating NK cells (18). Tumor immunoediting selecting variants with decreased expression of stress-induced ligands provide tumor escape to NK cell-mediated lysis through activating receptors NKG2D or NKp46 (19, 20).

The challenge is thus to overcome tumor immunosuppression and restore NK cell activities. To this aim, understanding the mechanisms that lead to NK cell defects in tumor is required.

## NK Cells in Hematological Malignancies

Numerous studies showed that severe quantitative and qualitative alterations of NK cells are associated with different hematological malignancies, particularly in myeloid disorders. In chronic myelogenous leukemia patients, low numbers of NK cells are associated with defects in their proliferation, and weak NK cell cytolytic functions in comparison with healthy donor blood NK cells (21). Furthermore, profound alterations in the activating receptors profile have also been reported including downregulation of NKp30 and NKp46 as well as DNAM-1, 2B4, and NKG2C on NK cells from acute myeloid leukemia (AML) patients. Decreased NKp30 and NKp46 expression was correlated with reduced NK cell killing and poor leukemia prognosis (22–25). Recently, Khaznadar et al. analyzed by cell imaging the lytic NK immunological synapse following interaction with AML cells and showed defective lytic granule polarization in NK cell-AML conjugates leading to impaired NK cell cytotoxic function (26).

Importantly, the intimate relationship between immune pressure and leukemogenesis has been suggested in two recent studies. Stringaris et al. described an immunoediting process induced by AML blasts that limits NK cell control of leukemia. They showed that abnormal NKG2A expression and TNFα production predict a poor response to chemotherapy in AML patients (27). Conversely, Khaznadar et al. showed that NK cell defects in AML patients at diagnosis could be associated with a specific transcriptional program in AML blasts and with patient’s outcome including relapse occurrence (28).

Furthermore, the beneficial role in the graft-versus-leukemia (GvL) of allogeneic NK cells for leukemic patients receiving allogeneic hematopoietic stem cell transplantation (HSCT) is well documented (29). Several studies showed that NK cells have a potent GvL effect in both KIR/HLA-class I-mismatched and -matched donor–recipient combinations after allogenic HSCT in AML patients (30–32). Moreover, rapid NK recovery after HSCT is also associated with a greater GvL effect and improved outcome in AML patients (33).

## NK Cells in Solid Tumors

*In situ* detection of NK cells infiltrating various human tumors/tissues was carried out, leading sometimes to divergent results due to the disparity of NK cell markers used (CD57, CD56, NKp46, double CD3/CD56 staining). However, several reports showed that NK cells can infiltrate clear-cell renal cell carcinoma (34), melanoma (35), non-small cell lung cancer (NSCLC) (36), breast cancer (BC) (37), GIST (38), and colorectal carcinoma (CRC) (39) although NK cells were mainly localized at the tumor’s periphery. In several tumors, infiltrations by NK cells were reported to have a prognostic value. Increased overall survival was associated with a high NK cell infiltrate within the tumor or tumor stroma in lung adenocarcinoma (40), metastatic renal carcinoma (41), and lung metastasis of renal cancer (42). Elevated number of NK cells was associated with reduced risk of cancer progression in prostate cancer (43), with a reduced risk of death in squamous cell lung cancer (44), and a better prognosis in gastric carcinoma (45) and CRC (46). In addition, the number of NKp46^+^ NK cells was found inversely correlated with metastasis occurrence in patients with GIST (47). Furthermore, a positive association between a high numbers of tumor infiltrating CD56^+^ NK cells with a regression of melanocytic lesions was observed (48).

In most tumor types studied, *ex vivo* tumor-infiltrating NK cells displayed severe phenotypic and functional alterations compared to blood NK cells and more interestingly compared to NK cells present in adjacent normal tissues. Those alterations affected the expression of activating receptors including NKp30, CD16, DNAM-1, and ILT2 on NK cells from patients with non-invasive and invasive BC (49) or NSCLC (36). A concomitant-increased expression of the inhibitory molecule NKG2A was also observed in BC (49). This deficient phenotype was associated with impaired functions including decreased cytotoxicity against tumor cells (36, 49) and reduced IFNγ production (36). Recently, Carrega et al. reported that lung and BC tissues were highly enriched in CD56^bright^perforin^low^ NK cell subset compared to matched normal tissues (37). It is of note that comparison between NK cells from tumor and normal adjacent tissue is required for better understanding of the effect of the tumor environment on their activation.

Interestingly, our team recently identified in tumor draining LN from melanoma and BC patients, the presence of a CD56^bright^CD16^+^ NK-cell subset that displays higher expression of activating receptors, perforin molecules, and performs ADCC (50). We found that different NK receptors regulate the two LN-NK cell subsets in melanoma and BC (personal communication) and that NK-infiltrating LN recapitulate the alterations reported in the primary tumors. The presence of CD16^+^ NK cells in certain tumors (51) and metastatic LN emphasizes the interest for ADCC function of such NK cells.

### Alterations in Blood NK Cells from Patients with Solid Tumors

Alterations in blood NK cells from patients with solid tumors were also reported, but in a lesser extent than in tumor infiltrating NK cells. Compared to healthy donors, a downregulation of NKG2D and an increase of the inhibitory receptor CD158b expression were correlated with impaired NK cell function (52–54) in metastatic melanoma patients. Our group showed a progressive decrease of NKp46 expression on blood NK cells with the disease progression in melanoma patients (55). In BC patients with invasive tumor, blood NK cells display altered expression of activating receptors NKp30, NKG2D, DNAM-1, 2B4, and CD16 and an upregulation of the inhibitory receptors NKG2A and CD85j. This phenotypic change was correlated with decreased NK cell cytotoxicity function and cytokine production (IFNγ and TNFα) (49). Blood NK cells from soft-tissue sarcoma patients displayed reduced proportions of CD56^dim^ NK cells. Low percentages of blood NK cells associated with a reduced NKp30, NKp46, and NKG2D expression were reported in patients with invasive squamous cervical cancer (56).

## NK Cells: A Potential Partner for Targeted Therapies

The advent of targeted therapies that counteract a vital cellular process within the tumor cell greatly improved cancer treatment strategies. Thus, mitogen-activated protein kinase (MAPK) inhibitors that control the mutation-driven oncogenic pathway present in most cancers are new efficient players in the arsenal of therapies for cancer patients. In addition, monoclonal antibodies (mAbs) that recognize tumor-associated antigens have been established as one of the most successful therapeutic strategies for both hematologic malignancies and solid tumors. These mAbs may activate antibody-dependent cell-mediated cytotoxicity involving NK cells.

Combining targeted therapies and methods to stimulate patient’s immune players is actively evaluated and represents a promising and natural evolution in cancer treatment as this could ally immediate efficiency, specificity, and long-term antitumor efficacy.

**Figure 1:**
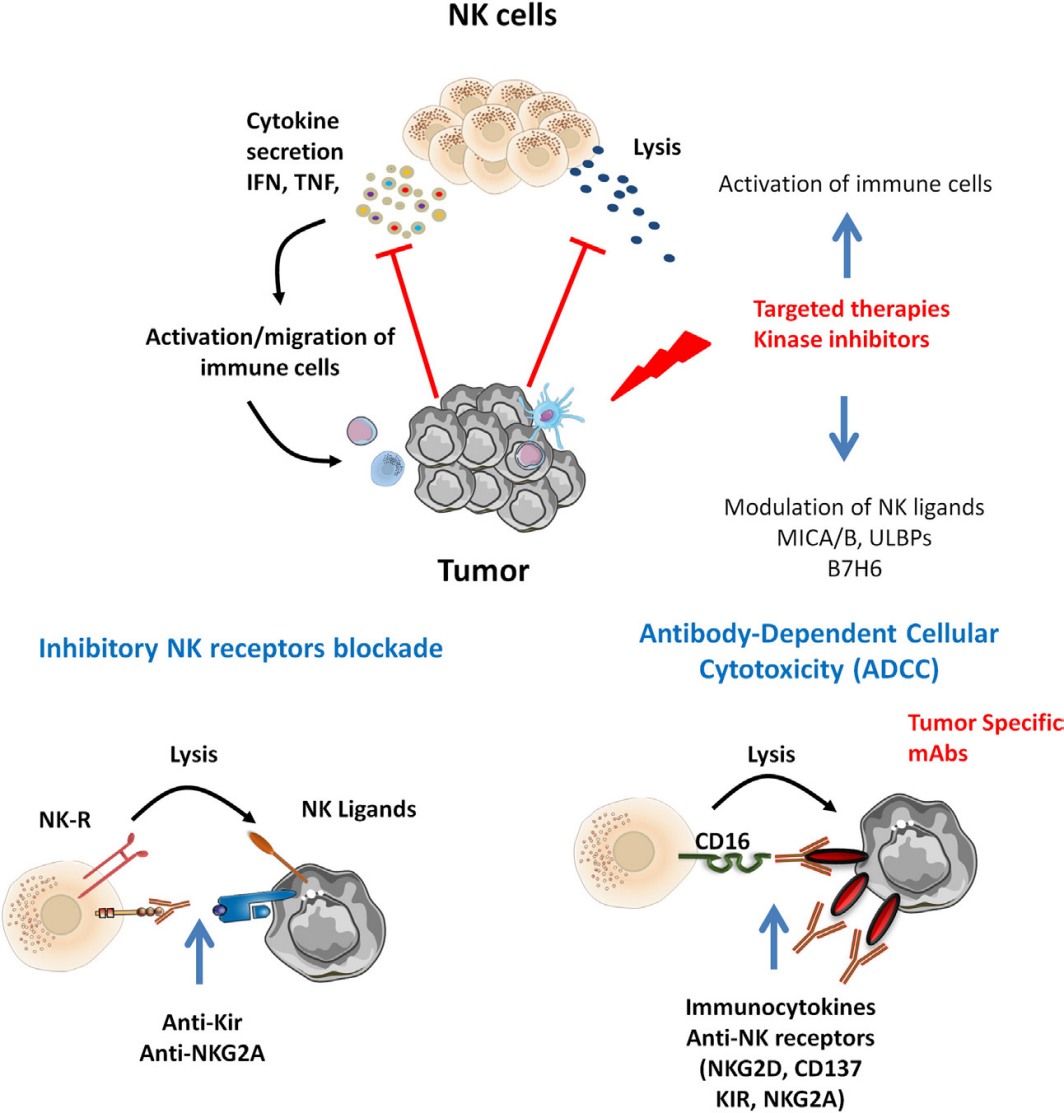
Natural killer (NK) cells infiltrating various tumor types display altered functions. Tumor-specific therapies may potentiate NK cell activation. Inhibitors targeting MAP kinase reduce tumor growth and display off-target effect modulating NK ligands expression and immune cell activation. Tumor-specific monoclonal antibodies (mAbs) may trigger ADCC by CD16^+^ NK cells. In that context, target NK-based immunotherapies may be proposed. Combined mitogen-activated protein kinase inhibitors with cytokines release NK function by inhibitory NK receptors [killer Ig-like receptor (KIR), NKG2A] blockade and promote NK-mediated ADCC of tumor antigen-specific mAbs and combined immunocytokines, anti-NK receptors (NKG2D, CD137, KIR, NKG2A).

It is of note that targeted therapies also display off-target effects, connecting oncogenesis to immunosurveillance. We discuss below the interest of NK cell-based therapies in the context of such tumor-targeted therapies (Figure 1).

### Effect of Cancer Treatment on NK Cells

Most melanoma patients (65%) bear a *BRAF*-mutated tumor and receive specific inhibitors targeting mutated *BRAF^V600E^* alone or in combination with MEK inhibitors, upstream of ERK (57). These inhibitors may exert bystander effects on certain immune cells that depend on MAPK for their activation and/or proliferation. BRAF inhibitors do not affect NK cell phenotype *in vivo* and *in vitro*, but blood NK cell numbers were increased in vemurafenib-treated patients (58, 59). MEK inhibition alters the expression of the main NK receptors and the function of cytokine-activated NK cells, but the combined BRAF and MEK inhibitors did not (60).

In addition, targeted therapies may interfere with the NK/target interactions through modulation of NK ligands on cancer cells. We have shown that a BRAF inhibitor modulates the expression of MICA and ULBP2 (ligands of NKG2D), changing the ratio between membrane expression and soluble form, and increases B7H6 (ligand of NKp30) expression and HLA-A,B,C and HLA-E molecules expression that engage inhibitory receptors (KIRs, NKG2A), thus interfering with NK cell-mediated lysis (in revision). Resistance to a BRAF inhibitor is accompanied by higher NK ligands expression (personal communications).

Our findings and recent results from the literature emphasize that therapeutics designed to limit cancer cell growth by acting through kinase inhibitors should also be considered in terms of their impact on immunosurveillance (61). In a murine model of *BRAF*-mutated melanoma, host NK cells and perforin were required for the effect of a BRAF inhibitor (62) and correlated with the reduction of tumor growth, and an increased NK and T cell infiltration of the tumors (63).

Combining specific MAPK inhibitors with immunotherapies to increase response rates is evaluated leading to yet discordant results. BRAF inhibition augments melanoma antigen expression and maintains T cell function (64). However, inhibition of BRAF in a murine model of human melanoma was associated with decreased tumor-resident lymphocytes and resistance to CTLA-4 mAb (65). MEK inhibitors increased antigen-specific T cell within the tumor sparing their cytotoxicity and combined with anti-PD-L1 mAb they exerted a synergic effect of tumor growth inhibition (66). Other kinase inhibitors such as those targeting Jak involved in the signaling cascade of cytokine receptors may influence NK (67).

A better understanding of off-target efficacy of MAPK inhibition affecting tumor–host interactions is required to develop strategies aimed at facilitating antitumor immune responses. The emerging findings indicate a potential synergy between targeted therapies, which change the balance between ligands of activating and inhibitory NK receptors, and NK-based immunotherapies, opening new interesting opportunities for the design of clinical trials.

### Anti-KIR/Anti-NKG2A mAbs: Increasing NK Function by Blocking Negative Signaling

One promising approach is to release NK cell function with anti-KIR or anti-NKG2A mAbs as NK cells are strictly controlled by receptors specific for HLA-class I molecules. Fully human anti-KIR mAbs, 1-7F9 mAb, and then lirilumab (recombinant version with a stabilized hinge) were generated (68). They prevent the binding of KIR2DL1, KIR2DL2, and KIR2DL3 receptors to their HLA-C ligands and blocking their inhibitory signaling. *In vitro* and *in vivo* studies showed that anti-KIR mAbs augmented NK cell-mediated lysis of HLA-C^+^ tumor cells, including autologous AML blasts and autologous CD138^+^ multiple myeloma cells (68–71). In addition, transient increases of TNFα and MIP-1β serum concentrations and CD69 expression on NK cells were observed from treated patients (72). In a clinical trial, Benson et al. showed that 1-7F9 mAb is safe in patients with multiple myeloma and enhances *ex vivo* patient-derived NK cell cytotoxicity against tumor cells (73).

Other immune receptors highly expressed by NK cells are in development, such as anti-NKG2A (monalizumab).

Targeting inhibitory pathways in NK cell/tumor interactions may be complementary to small-molecule inhibitors for the treatment of advanced tumors such as melanoma. The prospect of combining NK cell-based immunotherapy with approaches to target the immunosuppressive tumor microenvironment or immune checkpoints, such as KIR blockade, is especially relevant to the treatment of solid tumors (74, 75) and particularly for tumors refractory to targeted therapies.

### NK Cell-Mediated ADCC Using Tumor-Specific mAb

Natural killer cells express activating low-affinity FcgRIIIa (CD16) and are key mediators of antibody-dependent cellular cytotoxicity. The relevance of ADCC in tumor control using therapeutic mAbs was evaluated in several cancers. The contribution of ADCC to the clinical efficacy of a therapeutic mAb has been observed in non-Hodgkin’s lymphoma patients treated by anti-CD20 (rituximab) (76). Other therapeutic mAbs likely inducing NK cell-mediated ADCC are anti-CD19 in patients with B malignancies, anti-GD2 in neuroblastoma patients, and anti-HER2 mAbs (trastuzumab) in metastatic breast and gastric cancer patients (76–78). Anti-EGFR mAb (cetuximab) was shown to increase ADCC-mediated lysis of colon tumor cells by blood NK cells from colorectal cancer patients that display altered natural cytotoxic activity (51).

Several modifications of the antibody structure, such as class switching, humanization, and point mutations to reduce complement interaction/activation, are developed to engineer mAbs with increased NK cell ADCC function and limit their toxicity. Thus, humanized anti-GD2 mAb (hu3F8-IgG1) exerts reduced toxicity compared to other anti-GD2 mAbs, by leveraging ADCC over complement-mediated cytotoxicity (79). Higher FcγRIIIA-binding affinity of anti-CD19 antibody significantly increased NK cell-mediated ADCC, leading to malignant B-cell clearing in non-human primates (78, 80). Other strategies to enhance the effect of ADCC include the coadministration of cytokines, IL-12 with anti-HER2/neu (trastuzumab) (81) to stimulate IFNγ production by NK cells and T cells and promote the CD56^dim^CD16^+^ NK cell differentiation to mediate ADCC (82). Co-infusion of anti-CD20 (rituximab) and TLR9 agonist (CpG) that is known to raise the membrane expression of CD20 on malignant B cells enhances ADCC (83). The infusion of immunocytokines, cytokines linked to the Fc terminus of humanized Abs, is also evaluated to potentiate ADCC. In preclinical study, Buhtoiarov et al. demonstrated that the humanized anti-GD2 immunocytokine hu14.18-IL-2 exerts higher antitumor effect than the reagents given separately (84).

Combining tumor-specific mAbs and mAbs targeting NK receptors (NKG2D, costimulatory molecule CD137) is another option. Anti-CD137 coadministred with rituximab led to a subsequent stimulation of these NK cells and enhanced rituximab-dependent cytotoxicity against the lymphoma cells (85). Furthermore, combination of rituximab with antibodies that block KIR2DL1 significantly improved NK cell-mediated lysis of tumor targets (86).

## Conclusion

Restoring NK cell functions in addition to administration of tumor-specific therapies with kinase inhibitors or tumor-specific mAbs may benefit patients. It would increase the control of residual tumor cells, enhance mAbs efficiency, and promote the adaptive immune response necessary for long-lasting protective immunity. In that context, cytokines, blockade of inhibitory NK receptors (KIRs, NKG2A), or transfer of alloreactive NK cells are promising NK-based therapies.

## Ethics Statement

The study protocol was approved by an ethic committee “Ile de France” (CPP: 2834), and the Declaration of Helsinki protocols were followed.

## Author Contributions

MM, ND, and AC wrote the manuscript; MM did the figure; AF, PG, and AT read and corrected the manuscript.

## Conflict of Interest Statement

The authors declare that the research was conducted in the absence of any commercial or financial relationships that could be construed as a potential conflict of interest.
